# A Novel Method of Color Appearance Simulation Using Achromatic Point Locus With Lightness Dependence

**DOI:** 10.1177/2041669518761731

**Published:** 2018-03-16

**Authors:** Ichiro Kuriki

**Affiliations:** Research Institute of Electrical Communication, Tohoku University, Sendai, Japan

**Keywords:** color appearance, achromatic point, color appearance models, lightness

## Abstract

The purpose of the present study is to propose a simple algorithm for color appearance simulation under a color illuminant. Achromatic point is a chromaticity of rays that appear neither red nor green, neither blue nor yellow under a given illuminant condition. Saturation and hue of surface colors are evaluated with respect to the achromatic point of the same lightness, while the achromatic point under a colored illuminant depends on the lightness tested. We previously found that this achromatic point locus can be simply approximated as a line with a parallel offset from the lightness axis of CIE LAB space normalized to daylight. We propose a model that applies shifts in the lightness direction after applying hue/saturation shifts using the cone-response (von Kries) transformation under an iso-lightness constraint, such that achromatic points would be aligned with the lightness axis in the CIE LAB space under daylight normalization. We tested this algorithm, which incorporates evaluation of color appearance in different lightness levels, using #theDress image. Resemblance between our simulation and subjective color-matching results implies that human color vision possibly processes shifts in color and lightness independently, as a previous study reported. Changes in the chromaticity distribution of the images were compared with conventional models, and the proposed model preserved relative color difference better, especially at the lower lightness levels. The better performance in lower lightness levels would be advantageous in displays with wider dynamic range in luminance. This implies that the proposed model is effective in simulating color appearance of images with nonnegligible lightness and color differences.

## Introduction

Shifts in color appearance under different illuminant colors have been studied by many groups (Fairchild, 1998, 2001, 2013; Hunt, 1994; Luo & Hunt, 1998; Nayatani, Takahama, Sobagaki, & Hashimoto, 1990), and most of these works were based on the so-called von Kries-type transformation (von Kries, 1970), which applies gain changes to each cone type. Some studies proposed that the color appearances under chromatic illuminants are better explained by gain control, such that an achromatic surface (i.e., a spectrally nonselective surface) to be aligned to the subject’s *achromatic point* under a given chromatic illuminant (Kuriki, Oguma, & Uchikawa, 2000; Speigle & Brainard, 1999). The achromatic point is a chromaticity of a ray that appears colorless to an observer under a chromatic illuminant, and it is known that it usually does not coincide with the chromaticity of the illuminant because of incomplete chromatic adaptation (Fairchild & Reniff, 1995; Kuriki & Uchikawa, 1998; Kuriki et al., 2000; Speigle & Brainard, 1999).

The achromatic point was considered to be independent of the intensity of test stimulus, which means that the gain adjustment factors (“von Kries coefficients”) are invariant with the relative luminance of the test color with respect to the background, at the same time. On the other hand, several studies have pointed out that the achromatic point changes systematically with the relative luminance of the test color with respect to the background (Bäuml, 2001; Helson, 1938; Helson & Michels, 1948; Judd, 1940; Kuriki, 2006). The use of luminance dependence of the achromatic point locus improved the estimation of shifts in color appearance by 40% in ΔE ab* (Kuriki, 2015). However, the difficulty in describing the luminance dependence of the achromatic locus has been a problem when implementing to a color appearance model. Nayatani’s group made an attempt to implement this factor in the von Kries coefficients (Nayatani et al., 1990), but their idea was not adopted in the color appearance models that were proposed later (CIE CAM97s: Luo & Hunt, 1998; CIE CAM02: Moroney et al., 2002), mainly due to the complexity of formulating the achromatic point locus.

Our recent study proposed a new view to the dependence of achromatic point locus to luminance/lightness (the latter is proportional to the logarithm of the relative luminance of the test color with respect to the background). When the loci of chromaticity that *appeared achromatic* under various illuminant colors were plotted in a CIE LAB color space normalized to daylight D_65_ (in short, *CIELAB_D65_* in the followings), the achromatic point loci under various illuminant colors showed a systematic offset from the illuminant chromaticity (Chauhan et al., 2014; Kuriki, 2006). We found that the systematic offsets take the form of parallel lines from the lightness axis (a*, b*) = (0, 0) in CIELAB_D65_ (Figures 6 and 7 in Kuriki, 2015). Therefore, if this simple approximation were effective, it would significantly simplify the algorithm for the estimation of color appearance shifts under illuminant color changes.

The primary purpose of the present study is to propose a method to estimate the color appearance of surfaces under chromatic illuminants, by using our simple approximation of achromatic point locus (Kuriki, 2015). Note that the *achromatic surface,* which has a flat reflectance across the visible spectrum, *does not always appear achromatic* under chromatic illuminants due to incomplete chromatic adaptation (Fairchild & Reniff, 1995; Kuriki & Uchikawa, 1998). Hence, the *achromatic point* in this article is not always equivalent to *achromatic surface*.

Another issue to be argued on the von Kries-type models is not taking illuminant intensity changes into account. The excessive increase/reduction in chromatic saturation takes place in higher/lower lightness range. This occurs when using a single set of von Kries coefficients derived with target illuminant of different intensity because most color appearance models are designed to simulate shifts in color appearance under illuminant *color* changes but substantial intensity changes. Considering the case of shifts in intensity together with color, use of a single set of von Kries coefficients will evoke over saturation and desaturation in color appearance under illuminant intensity changes; this will be shown later in this article. The present study also proposes a method of color appearance simulation, which includes compensation for nonnegligible amount of illuminant intensity differences.

In the proposed model, color shifts were estimated by applying achromatic point shifts using von Kries coefficients (Kuriki, 2000; Speigle & Brainard, 1999) estimated in the same lightness (L*) so that the achromatic point aligns with the lightness axis, where the coefficients varied with the lightness of the color to be transformed (Kuriki, 2015). After color shifts were calculated within each lightness level of the test color, a lightness shift was applied by simply shifting L* values such that the dress-body or lace part would appear white or black, respectively. This process is necessary when treating images that incorporate evaluation of color appearance in different lightness levels, for example, #theDress image; however, not much is explicitly written in the previous models. Finally, we report that images generated by the proposed method show a better estimation of human subjects’ matches using #theDress image as an example.

## Model Description for Color Appearance Shifts

### Luminance Dependence of Achromatic Point Loci

**Figure 1.**
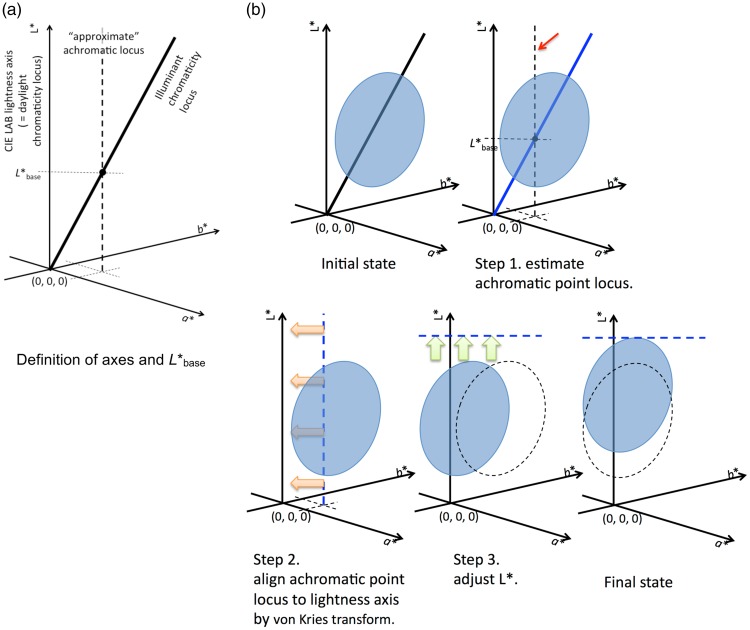
Schematic diagram of color shift simulation in CIE LAB_D65_ space. (a) Illuminant chromaticity locus does not coincide with lightness axis of CIE LAB space, normalized to D_65_ (CIE LAB_D65_). Approximate achromatic locus is derived with L base*. (b) Shaded ellipses represent gamut of an object in an image. Step 1: estimation of achromatic point locus, Step 2: alignment of achromatic point locus to lightness axis, and Step 3: adjustment of lightness differences. See text for details.

As reported in previous studies, achromatic locus, which is a locus of chromaticities that appear colorless at each lightness under a given illuminant, does not coincide with illuminant chromaticity. In most cases, reflected light from achromatic surfaces (e.g., white or various shades of gray papers) is very close to illuminant chromaticity. However, they do not always appear achromatic, due to incomplete adaptation (Fairchild & Reniff, 1995; Kuriki & Uchikawa, 1998; Kuriki et al., 2000; Speigle & Brainard, 1999). The achromatic point locus also has an important feature that it is lightness dependent (Helson & Michels, 1948; Kuriki, 2015). An achromatic point *appears* achromatic under a given illuminant. Therefore, in a color space, which represents subjective color appearance under an illuminant, an achromatic locus should be aligned to the point of zero chromaticity, that is, achromatic axis. In the present study, we use a *CIELAB_D65_* (normalized to D_65_, 100 cd/m^2^) as the space for the simulation of color appearance (Kuriki, 2015; Figure 1(a)).

### Outline of the Model

There are three major steps: (a) estimation of achromatic point locus from image, (b) apply chromaticity shift in each lightness plane, and (c) apply lightness shift. The details are explained one by one as follows, and the steps are illustrated in Figure 1. Also, formulations for these steps are described in Appendix.

Step 1. Estimation of locus of achromatic point

As described in details in the previous study, the achromatic point locus seems to coincide with the locus of illuminant chromaticity in CIELAB_D65_ space at a relatively low lightness level: around L* = 20∼35. Therefore, once illuminant chromaticity is given, an approximate of achromatic locus that should be aligned to (a*, b*) = (0, 0), that is, lightness axis of CIELAB_D65_ space could be estimated by this rule. See *Step1* in Figure 1. In the present study, L* = 25 will be used as L base*, based on our previous study (Figure 7 in Kuriki, 2015).

Step 2. Application of chromaticity shift

As suggested by previous studies (Kuriki, 2015; Kuriki et al., 2000; Speigle & Brainard, 1999), the alignment of achromatic points to L* axis of CIELAB_D65_ space by von Kries transformation works as a good estimate of shifts in color appearance. In addition, if this transform is applied in each L* level, due to the difference in “von Kries coefficients” to different lightness levels, the approximation fits the apparent color shifts improves (Kuriki, 2015). Therefore, von Kries coefficients were derived for each point of image based on its L* value, and then von Kries transformation will be applied (Figure 1(b)).

Step 3. Application of lightness shift

In most color appearance models, shifts in lightness are not implemented (Fairchild, 1998; Hunt, 1994; Nayatani et al., 1990; CIE CAM02) because they are designed to compensate for illuminant chromaticity, without drastic change in illuminant intensity. However, considering an application of such color appearance model to images that have to be compensated for the intensity, as well, this factor has to be taken into account. In a previous study, changes in hue and saturation of color surfaces were tested between various illuminant intensities (Newhall, Burnham, & Evans, 1958). This implies that the compensation for illuminant intensity may be approximated by shifts in lightness level. This step is shown in *Step 3* in Figure 1.

This step would be applied, when evaluation of color appearance in different lightness levels is incorporated, so that a particular surface in the image to be either black or white, if they were perceived as so. #theDress image is one of the examples so that either the dress-body part appeared white or the lace part appeared black, under the extreme cases.

### The Treatment of Spatial Context Factors

It is known that spatial context also affects color appearance; for example, a neutral gray patch appears colored when surrounded by an area with a saturated color. Our model does not implement any factors by spatial context. It means that the (a*, b*) values *alone* in the output image do not directly represent color “appearance.” It is nearly impossible to completely quantify the effect of, for example, simultaneous color contrast in any form. Our model represents the compensation for shifts in illuminant color changes only and allows the spatial effect to be applied within each observer’s visual system; this theory is already adopted in RLAB model (Fairchild, 1998). But the absence of this compensation becomes visible when an asymmetric color matching is made by adjusting the appearance of a color chip in an isolated space, that is, in a dark background. This will be pointed out again in a later section.

## Test With #theDress Image

### Purpose and Methods

To demonstrate the efficacy of our model, we used a color-matching data for #theDress image, for which a huge variety of individual difference in color appearance is reported (Brainard & Hurlbert, 2015; Gegenfurtner, Bloj, & Toscani, 2015; Hesslinger & Carbon, 2016; Lafer-Sousa, Hermann, & Conway, 2015; Winkler et al., 2015; Witzel, Racey, & O'Regan, 2017). As reported in a recent study, the individual difference was represented as the color and intensity of illuminants “implicitly assumed” in each subject (Witzel et al., 2017). Particularly, one of the two major causes of individual difference was the assumption of illuminant intensity, which is suitable to test the efficiency of lightness adjustment in the proposed model. Also, this image consists of two major parts (dress-body and lace parts), which simplifies evaluating the appropriateness of the model. Furthermore, the color-matching data of color appearance in 15 individuals, together with their descriptive perception (blue/black or white/gold), are available in public. Since data were provided by other research group, the data were not collected on our favour. It also enables to perform evaluations by other groups. Therefore, we will demonstrate our model estimates color appearance with this #theDress image. All image processing and analyses in the following were conducted on MATLAB 2016b (Mathworks Inc., MA, USA). The result of estimation would be represented as an image in CIE LAB_D65_.

**Figure 2.**
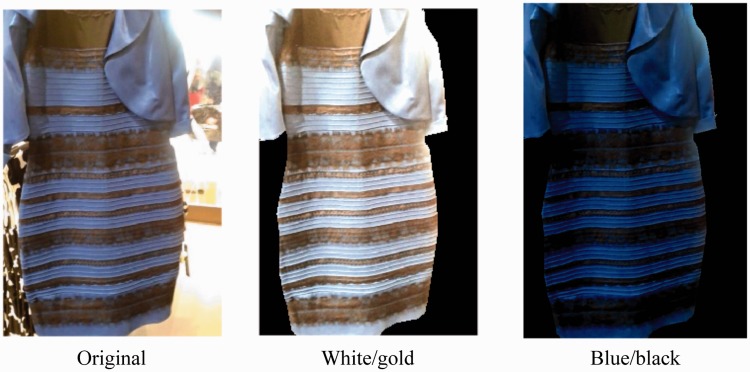
Simulated “white/gold” image (center) and “blue/black” image (right). The proposed method was applied by using an achromatic locus approximated from a mean of dress-body colors under L base* = 25. High intensity (background) was excluded when generating color appearance simulations (right two images). See main text for details.

To confirm whether the approximation shown in Step 1 of Figure 1(a) also applies to #theDress image, the achromatic point loci were assumed to be a line parallel to the lightness axis, under a given L*_base_ to calculate statistics. Images generated by using mean CIE a*b* chromaticity of dress-body part under L base* = 20 or 30, based on our previous study (Figure 7 of Kuriki, 2015), appeared “white/gold” colors. For the present study, L base* = 25 was used (Figure 2, center). Another figure was generated by applying the same algorithm so that the mean chromaticity of lace part would be black (L* = 3 was used as an attempt); this yields a “blue/black” image (Figure 2, right). In these cases, the von Kries coefficients were generated for each lightness level so that the achromatic locus estimated from either the dress-body part (white/gold: center) or lace part (blue/black: right) of the image becomes achromatic (a*, b*) = (0, 0).

### Simulating Individual Differences

**Figure 3.**
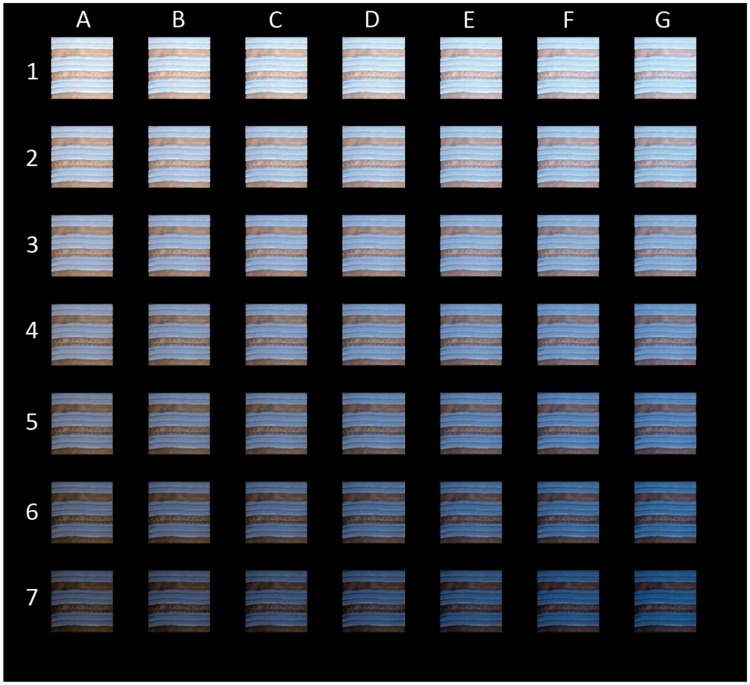
Various appearance estimates. The most “white/gold” pattern is at the top-left (A-1: Figure 2 center), and the most “blue/black” pattern is at the bottom-right (G-7: Figure 2 right). The top row is based on the darkest illuminant assumption, and the bottom row is based on the brightest illuminant assumption. The leftmost column images are based on the estimation that dress-body is achromatic (blue illuminant), and rightmost column images are based on the estimation that lace part is achromatic (white illuminant). The original image is closest to the one in the center (D-4).

Previous studies reported the presence of variability in the appearance (see Figure 1(b) and (c) in Gegenfurtner et al., 2015; Lafer-Sousa et al., 2015), and it is also reported that the “assumed (implicitly)” illuminant can be different among individuals in both color and intensity (Witzel et al., 2017). Therefore, additional images were generated by assuming the intermediate color/intensity levels of *assumed* illuminants between the two extremes. Figure 3 shows variants of dividing the difference between the “white/gold” and “blue/black” extremes into six steps. Images A-1 to G-7 are derived by varying parameters of illuminant color and intensity for the model estimation, in equal steps in the CIELAB_D65_ space between the case of the bluest and darkest estimate (A-1) and the whitest and brightest estimate (G-7) of the illuminant.

When the color and illuminance parameters in each individual are estimated independently, some variants may fall off the diagonal line connecting “A-1” to “G-7.” The appropriateness of the proposed method may be confirmed by the presence of such off-diagonal cases, and this point will be assessed by comparisons with the results of color-matching data for the color appearance of the dress-body and lace parts.

### Comparison With Actual Color-Matching Data

**Figure 4.**
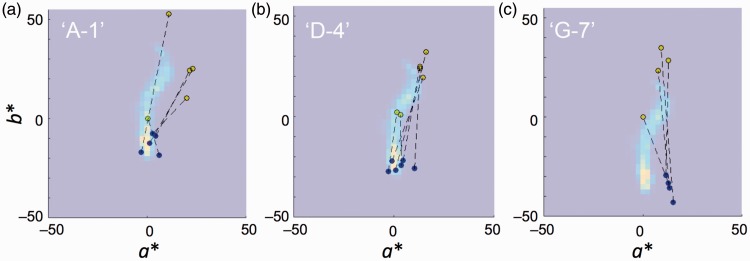
Comparison between generated images and matching results in a*b* plane. Panels (a) to (c) represent 2D histogram for images “A-1,” “D-4,” and “G-7,” respectively. Symbols represent color-matching results from Gegenfurtner et al. (2015) in the a*b* plane. Blue and yellow symbols, paired with dotted lines, are matching results for the dress-body and lace parts, respectively.

To evaluate the efficiency of our model, the output images were compared with color appearance data from other group (Gegenfurtner et al., 2015); the use of data from other group is aimed to remove the possibility that the data were collected in favor of our concept. A data set of color matching for the dress-body and lace colors, and this data were used for the comparison with a histogram of chromaticity in the generated image. Figure 4 shows the superposed plot of chromaticity histogram (background) and color-matching data (round symbols). Histograms represent the chromaticity distribution of the whole dress image (excluding background) for “A-1,” “D-4,” and “G-7” in Figure 3. The assignment of individual data to one of three groups was arbitrarily decided for the purpose of showing the results at a glance, instead of showing all panels of each participant’s data.

Different shades of the background represent population of the pixel chromaticity, binned at 2.5 steps in a* and b* coordinates of CIELAB_D65_ space. Bright spots in Figure 4(a) to (c) represent the chromaticity of the dress-body part, and another modal population at the middle or above represents the cluster each corresponding to the lace part. A pair of filled circles (blue and yellow), connected with dotted lines, represents each participant’s subjective matching result from a previous study (Gegenfurtner et al., 2015). Symbols filled with blue and yellow represent the results of matches to dress-body and lace parts, respectively. The RGB values were transformed to XYZ tristimulus values by using the information of color primaries described in their text, and CIE Lab coordinates, normalized to D_65_, were calculated.

To make the comparisons easier, subjects (*N* = 15) were arbitrarily grouped by three levels (Group 1: Subject Nos. 2, 3, 7, 13, and 15; Group 2: Nos. 6, 8, 9, 11, 12, and 14; Group 3: Subjects Nos. 1, 4, 5, and 10; subjects are numbered by the order from top in the Table S1 in Gegenfurtner et al., 2015) based on the bluishness (b* value) for colors matched to the dress-body. The distribution of matching results for the dress-body part (blue symbols) is close to the highest value of the histogram (brightest spot), except for some deviation in the horizontal (a*) direction in Figure 4(c).

Since the chromaticity distribution of Figure 4(b) (D-4 in Figure 3) roughly corresponds to the color distribution of the original image excluding background, the results of color matching seem to show overestimation of yellowness in the lace part. This may indicate the presence of color induction to the narrow lace part from the surrounding bluishness of the dress-body area, due to chromatic contrast. The reason for slight deviations in the positive a* direction (reddish) in Figure 4(c) will be discussed later.

**Figure 5.**
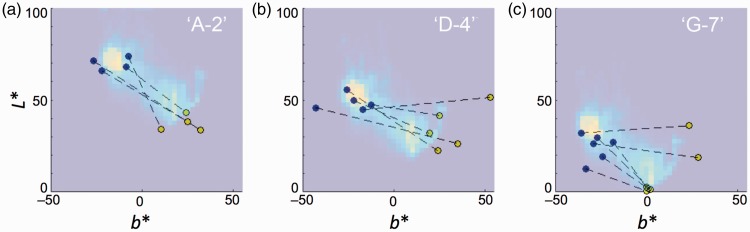
Comparison between generated images and matching results in b*L* plane. Shading of background represents the chromaticity histograms of images shown in Figure 3. Panels (a), (b), and (c) show the histogram of panels “A-2,” “D-4,” and “G-7,” respectively. The best matches for each subject were chosen by L* values for the dress-body part (blue symbols).

Figure 5 shows the same comparison in the b*L* plane. In this case, the groups used for the view in a*b* plane were not optimal; Group 1: Subject Nos. 2, 9, 14, and 15; Group 2: Nos. 4, 6, 7, 12, and 13; Group 3: Nos. 1, 3, 5, 8, 10, and 11. Such differences in the optimal groups between Figures 4 and 5 imply that the color and intensity estimation for the illuminant is independent, which is in line with the report by Witzel et al. (2017).

**Figure 6.**
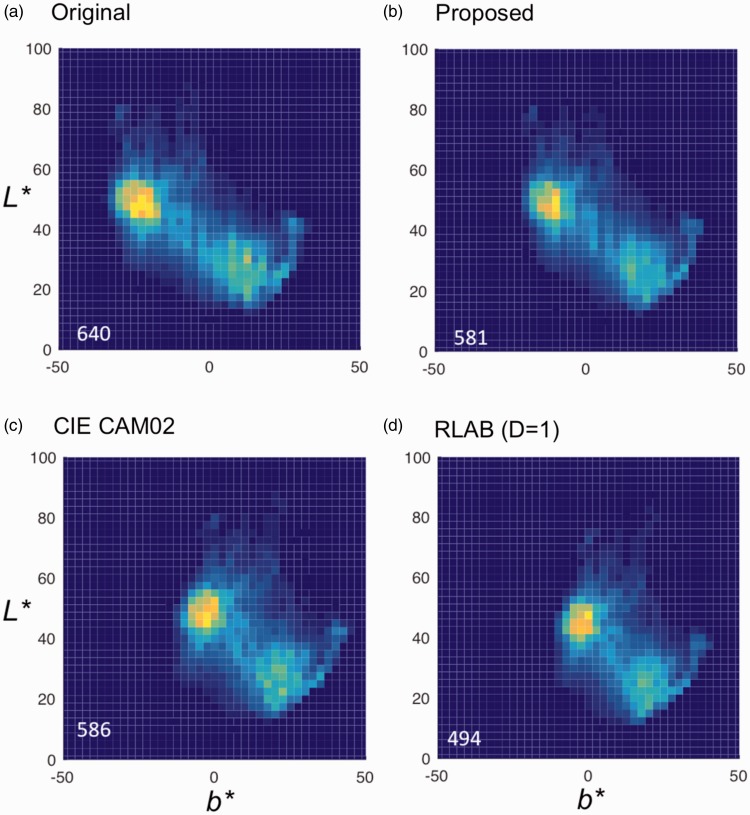
Chromaticity distribution of images processed by each method. Heat maps show population of pixels in each chromaticity in the b*L* plane. The numbers at the bottom-left corner indicate number of cells (binned at 2.5 × 2.5 in b* and L* values) with nonzero values. CIE CAM02 (c) shows a slightly larger number than our method (b), but the gamut is clearly shrunk horizontally, as is that in RLAB (d). See also Table 3 for distance of the two modal points (dress-body and lace parts) in each distribution.

Panels from Figure 3, whose histograms are shown as shadings in the background, were chosen so that they roughly match in L* level of blue symbols (dress-part matches) for a better visibility and conciseness of the figure. In most subjects, the distribution of yellow symbols (lace-part matches) also shows good agreement with the generated image with just one or two exceptions in each panel.

Considering the independent manipulation for hue/saturation and lightness (L*) level in our method, the *best match* appearance are found among those in Figure 3 panels for each participant. Table 1 shows the best matches chosen by the proximity between the modal point of the histogram (brightest spot) and the matched color of the dress-body part (blue symbol) for “White-Gold” person (Subjects #2, 4, 6, 7, 9, 10, 12, 13,14, and 15) and lace part (yellow symbol) for “Blue-Black” person (Subjects #1, 3, 5, 8, and 11) because our algorithm calculates based on an assumption that each subject assumes an illuminant based on either of these two parts to appear achromatic (white or black).

**Table 1.** Best Matched Panel in Figure 3 for Each Subject.

Sub.	Panel no.	Sub.	Panel no.	Sub.	Panel no.
1	E-7	6	F-5	11	E-7
2	A-3	7	B-6	12	D-5
3	F-7	8	E-7	13	A-6
4	G-6	9	D-3	14	G-3
5	B-5	10	G-7	15	A-3

To examine the efficiency of the image estimation, residual color difference in ΔE LAB* was examined by comparing distances between subjects’ matches and optimal image (Table 1) for each subject. The residual color differences was tested in the remaining part that might appear chromatic (i.e., *yellowish* lace part for white/gold type and *bluish* dress-body part for blue/black type) because the optimal pair was found by the least error for the “achromatic” part of the matched results. To justify the efficiency of the model, residual color differences for randomly chosen pair for 15 subjects were calculated as a reference distribution. This calculation was repeated for 10,000 times to obtain the reference distribution of the residual error in randomly chosen pairs. The resulted residual difference for chromatic part of the optimal pair was 24.4 ± 2.30 (*N* = 15) in mean ± *SEM* in ΔE LAB*, whereas the reference distribution’s mean was 33.4 and 95% confidence interval was between 27.4 and 39.5 in ΔE*_LAB_. Therefore, the residual difference of the optimal pair (Table 1) is significantly smaller than the randomly chosen pairs (*p* < .05). The residual color differences of the “chromatic” part for two phenomenal type of subjects were 17.1 ± 4.82 (*N* = 5) and 28.1 ± 4.28 (*N* = 10) in mean ± *SEM* in ΔE*_LAB_ for blue/black and white/gold type, respectively. The reason of larger deviation for the lace part in the white/gold-type subjects will be discussed later in detail in the Discussion section.

The residual color differences for the “achromatic” part, by which the optimal pair was found, were 9.36 ± 1.41 for all subjects (*N* = 15) in mean ± *SEM* in ΔE*_LAB_; 11.3 ± 0.65 for blue/black type (*N* = 5), and 8.39 ± 2.01 for white/gold type (*N* = 10). This error for the “achromatic” part could be minimized by generating the intermediate images in Figure 3 with much finer steps in the illuminant color and intensity parameters. See Table 2 for the summary.

**Table 2.** Residual Error: Mean (*SEM*) in ΔE LAB*.

	All (*N* = 15)	Blue/Black (*N* = 5)	White/Gold (*N* = 10)
Chromatic part	24.4 (2.30)	17.1 (4.82)	28.1 (4.28)
Achromatic part	9.36 (1.41)	11.3 (0.65)	8.39 (2.01)

*Note*. SEM: standard error of the mean.

Also, the result shows that there is no significant correlation between the column and row numbers of Figure 3 (*r*^2 ^= .210; n.s.), and it implies that chromaticity (bluishness–yellowishness) and the intensity of *estimated* illuminant are independently chosen when evaluating surface colors in the image (Witzel et al., 2017).

### Comparisons With Previous Models

To compare the results with other color appearance models in terms of the quality of color shifts, two models were tested: CIE CAM02 (Moroney et al., 2002) and RLAB (Fairchild, 1998). Both models use a single set of von Kries coefficients under an illuminant color change. To equate conditions, the *chromaticity of the “estimated” illuminant in our method* was used as a normalization chromaticity; it was derived to make the dress-body achromatic (“white/gold” type) under L base* = 25. For the RLAB model, a parameter for hard copy (D = 1) was used because a parameter for soft copy (D = 0) did not discount bluish appearance by an assumed “illuminant” sufficiently. To focus on the characteristics of color shifts, L* shifts were not applied here for all models.

Chromaticity distribution profile in b*L* plane differed noticeably between the models. Figure 6 shows 2D histograms of b*L* values of the original (Figure 6(a)) and processed images by three methods (Figure 6(b) to (d)).

To make a quantitative comparison, chromatic contrast was defined by the distance between the two modal points that correspond to the dress-body and lace parts of #theDress image. Table 3 shows distance between two modal points of histograms in b* and L* coordinates. The von Kries-type models with the coefficients that do not depend on lightness or luminance (CIE CAM02 and RLAB) show reduction in color contrast in the b* direction, while our method showed minimal difference. Difference in the extent of chromaticity distribution in the b* direction, that is, horizontal displacement from zero, is more evident in the *darker* lightness range (i.e., the lace part) of Figure 6(c) to (d). The color shifts in our method (Figure 6(b)) were applied by the von Kries transforms to align the achromatic point locus to the lightness axis (Steps 1 and 2 in Figure 1(b)). This process may have worked to preserve color contrast/difference especially at the lower lightness levels.

**Table 3.** Contrast of Images in b*L* Plane.

Methods	ΔE b*	ΔE L*
Original image	43.8	27.0
Proposed method	38.5	30.0
CIE CAM 02	30.3	25.5
RLAB	23.0	27.3

## Discussion

The present study tested a color shift algorithm that simulate color appearance by three simple steps: (a) the estimation of the achromatic point locus as parallel to the lightness axis with an offset, (b) color shifts are applied by the von Kries transform by aligning the achromatic point to the L* axis in each lightness, and (c) a parallel shift along the L* axis to adjust lightness levels (Figure 1). The images generated by this method appear to work for the shift in color appearance under illuminant changes.

Each of these simple steps is supported by psychophysical studies. The hue/saturation shifts used an algorithm called the “Speigle–Brainard conjecture” (Schultz, Doerschner, & Maloney, 2006; Speigle & Brainard, 1999) with an extension to implement the dependence of the achromatic point on lightness level by a simple assumption. Shifting the (a*, b*) coordinates of the CIE LAB space in Step 2 (Figure 1) is much simpler than using a von Kries-type transformation, but this procedure has not been justified in a literature as a method to compensate for the color appearance by aligning the achromatic point locus. The efficiency of the “Speigle–Brainard conjecture” was confirmed also by our previous study using different experimental conditions (Kuriki et al., 2000), and its accuracy was improved by 40% in ΔE ab* when the luminance dependence of the achromatic point was applied additionally (Kuriki, 2015). As illustrated in Figure 6, the shrinkage/expansion in chromatic saturation at a darker/lighter part is a characteristic inherent to the von Kries-type transformation. Our method applied color shifts by cone-response adjustment in each lightness before applying an L* shift, and the desaturation/oversaturation problem is far less obvious. Changes in apparent hue/saturation are known to be relatively small when the reduction in reflected light intensity is due to moderate changes in illuminant strength (Newhall et al., 1958).

The results of our method were able to demonstrate variations in color-matching data reported in a study by a different group (Gegenfurtner et al., 2015). The original study mentioned that the lightness and color estimates of illuminant are correlated and lightness difference was more evident than color (bluishness) difference between individuals, but the present study supported the possibility that the illuminant color estimation and illuminant intensity estimation are rather independent (Witzel et al., 2017). The optimal estimations of illuminant chromaticity and intensity in each subject were not entirely correlated. As shown in Table 1, the independent manipulation of color (hue/saturation) and lightness in the proposed method was able to account for the result of individual differences in subjects’ appearance of the dress.

It must be clarified that our method does not implement color shifts due to factors other than illuminant color/intensity differences. This stance is the same as that of RLAB (Fairchild, 1998). The need of the other factors depends on the purpose of the appearance model to be used. One factor that was not included in our method is the effect of spatial-context effects such as chromatic induction. The matching results for the lace part (yellow symbols in Figures 4 and 5; Gegenfurtner et al., 2015) are considered to deviate in slightly higher saturation because the matched color of the lace part exceeded the chromaticity distribution of the original dress image, even for subjects that matched the dress-body part with almost the same chromaticity as the original dress image (Figure 4(b)). Such adjustments of chromatic saturation in lace part may be due an enhancement in yellowness induced by the surrounding blue dress-body region that has larger width/area than the lace parts; that is, chromatic induction. Such an effect by local spatial contrast on color appearance is always included when the observers view the picture using their own eyes (Figures 2 and 3). In fact, the magnitude of residual shift for optimal pairs (Table 1) in +b* (yellowish) direction for the lace part in white/gold-type subjects (19.8 ± 3.67 in mean ± *SEM* in ΔE*_LAB_; *N* = 10) was much larger than those in a* direction (8.39 ± 1.26; *N* = 10) or residual shift for dress-body part in blue/black-type subjects in a* (7.37 ± 2.08) and b* (5.90 ± 1.97; *N* = 5) directions. Therefore, the magnitude of residual difference of white/gold-type subjects and deviation of color matches for the lace part (Figure 5) could be due to color contrast, that is, the spatial-context effect, by the difference in areas of dress-body and lace parts.

Another factor that could have affected in the matching results, and that is not implemented in our method, is shifts in color appearance inherent to asymmetric matching. In any case of color matching, subjects have to first memorize a color to be matched before starting adjustments (however short it is), and a shift toward typical/ideal color for each subject could take place during adjustments. Therefore, increases of chromatic saturation in memory (Amano, Uchikawa, & Kuriki, 2002) could more or less affect the result of color matching. The shifts in the +a* direction for some subjects (Figure 4(c)) took place, possibly because they tried to reproduce as deep a blue as possible at the time of adjustments, in addition to that the longer wavelength components are known to contribute to bluishness appearance (Ejima & Takahashi, 1985).

CIE CAM02 is an improved version of a color appearance model defined by CIE (CIE CAM 97 s; Luo & Hunt, 1998), which is an integrated model of Hunt's (1994), Nayatani et al.'s (1990) and Nayatani (1995) models. RLAB is another color appearance model, specialized for color shifts in image processing, under a concept of applying minimal modification to the CIE LAB. These models are optimized to fit the results of color-matching experiments measured with color chips under illuminant color changes between illuminants A and D (Fairchild, 2013); both of them are based on the von Kries model with luminance-invariant coefficients.

The negatives of using luminance-invariant coefficients, as the ordinary von Kries model does, appeared in the smaller chromatic gamut in lower lightness colors. Previous studies about the lightness dependence of achromatic (equilibrium) points (Bäuml, 2001; Kuriki, 2006) and achromatic surfaces (Helson & Michels, 1948) under chromatic illuminants suggest that the achromatic point becomes more and more diverted away from illuminant chromaticity as the lightness goes down, as illustrated in Figure 1(a) as an “approximated” achromatic locus. Imagine that the chromaticity of light reflected by gray scale, that is, *achromatic surfaces*, would be aligned on the oblique line (“illuminant chromaticity locus”) and its relative location for lighter surface is more apart in the direction of illuminant color with respect to the achromatic point, and vice versa. This implies that lighter achromatic surfaces appear similar to the color of illuminant and darker surfaces appear more in the opposite color of illuminant; this is analogous to the Helson-Judd effect. The achromatic point locates relatively closer to lightness axis than illuminant chromaticity at higher lightness, and vice versa, in Figure 1(a).

This means that the von Kries coefficients become smaller than 1.0, when they are defined for the lighter surfaces to appear achromatic. Hence, colors at lower lightness change only slightly for von Kries models with lightness-invariant coefficients, and this is clearly the case in Figure 6(c) and (d). On the other hand, the proposed method uses color shifts based on the location of achromatic point under a given lightness level (Figure 1(a)). Therefore, achromatic point would be able to be achromatic. For the replication of blue/black-type appearance of the dress, the rendering of darker range with our method has clear advantage (Figure 2). This kind of difference may become more prominent in near future when displays with wider (higher) dynamic range (e.g., luminance range of 1.0 × 10^6^ or more), which can render darker area more vividly than those of the standard dynamic range (around 1.0 × 10^3∼4^), become more popular.

To conclude, the model proposed in the present study has clear advantage in rendering colors of surfaces in wider range of lightness in terms of chromatic saturation, especially in lower lightness levels.

## Appendix

### Formulation of the Color Appearance Transform

The color appearance transform was applied by following two major steps: (a) a color shift was applied within each lightness, based on the dependence of the achromatic locus on *L**, (b) the lightness was adjusted afterward. The lightness adjustment was not investigated in our previous study (Kuriki, Oguma & Uchikawa, 2000; Kuriki, 2015), but it became necessary to simulate differences in the estimated illuminant intensity to visualize white/black. In the present study, it was implemented, as an attempt, by simply shifting the *L** values. The *L** shift was chosen because it was reported that the light intensity does not vary hue and chromatic saturation drastically when a change in illuminance takes place between moderate lightness levels (Newhall et al., 1958). The variance of illuminant intensity estimation can be estimated to range no larger than 3 log_10_ units because the original dress image, the only information source to the observer, cannot hold luminance dynamic range larger than 3 log_10_ units.

The procedure for estimating shifts in color appearance based on the offset of the achromatic locus is described as follows. First, the illuminant color and intensity for the shift was estimated from two parts in the image. An ensemble representative color (statistics) of the dress-body (excluding lace part) textile or the lace part (excluding dress-body) was derived. Second, the chromaticity of the achromatic locus in the CIE LAB space was determined for the chromatic illuminant as a line parallel to the lightness axis with an offset in the direction of illuminant chromaticity (Figure 1).

(A1) $$\begin{array}{c} a_{\text{i}}^{*}=a^{*}(\text{illum},L_{\text{base}}^{*})\\ b_{\text{i}}^{*}=b^{*}(\text{illum},L_{\text{base}}^{*}) \end{array}$$

where $L_{\text{base}}^{*}$ represents a lightness level from which the illuminant chromaticity is adopted as the approximate “offset” of the achromatic locus (see Figure 1), and the achromatic locus chromaticity (*a**_i_, *b**_i_) under a given illuminant *i* does not depend on the lightness.

Third, a triplet of scaling factors for the cone responses (von Kries coefficients) was defined for the bluish/yellowish illuminant within each lightness level (approximated by *L** level) before applying the von Kries transformation (Kuriki, 2015; Kuriki et al., 2000; Speigle & Brainard, 1999). The derivation of coefficients was as follows:

(A2) $$k_{\{\text{L},\text{M},\text{S}\}}(L^{*})=E_{\left\{\text{L},\text{M},\text{S}\right\}}({\text{ach}}_{2},L^{*})/E_{\left\{\text{L},\text{M},\text{S}\right\}}({\text{ach}}_{1},L^{*})$$

where *k*_{L,M,S}_(*L*^*^) represents von Kries coefficients for L, M, or S cone for a lightness *L**, and *ach*_i_ (*i* = 1 or 2) represents the achromatic locus. Cone excitations *E*_{L,M,S}_ can be approximated by transforming *L***a***b** coordinates to CIE tristimulus value XYZ before applying cone fundamentals. In this study, the Smith–Pokorny (1975) cone fundamentals were used for simplicity. In the present case, *i* = 1 corresponds to, for example, blue or yellow, and *i* = 2 corresponds to daylight (i.e., the lightness axis). Let us assume the color appearance represented in a CIE LAB space normalized to the daylight D_65_ to follow the definition of the sRGB standard. See Figure 1(a) for a schematic diagram to explain the relationships between these parameters.

The shift in image chromaticity was conducted by applying a von Kries-like transformation of cone responses by using coefficients *k*_{L,M,S}_(*L**) under a given lightness level *L**, derived from the deviation of achromatic locus in Equation (2) (Figure 1(b)). Cone responses (*E*_Lc_, *E*_Mc_, *E*_Sc_) for a given chromaticity of a pixel *c* (*L*_c_*, *a*_c_*, *b*_c_*) were derived by transforming the CIE LAB coordinates through CIE tristimulus values (X, Y, and Z) before applying the cone fundamentals of Smith and Pokorny (1975). The new cone fundamentals after the color shift would be derived as follows:

(A3) $$\begin{array}{c} {}[\begin{array}{c} E_{\mathrm{LC}}\\ E_{\mathrm{MC}}\\ E_{\mathrm{SC}} \end{array}{]}_{\mathrm{new}}=[\begin{array}{ccc} k_{L}(L_{C}^{*}) & 0 & 0\\ 0 & k_{M}(L_{C}^{*}) & 0\\ 0 & 0 & k_{S}(L_{C}^{*}) \end{array}][\begin{array}{c} E_{\mathrm{LC}}\\ E_{\mathrm{MC}}\\ E_{\mathrm{SC}} \end{array}{]}_{\mathrm{org}} \end{array}$$

The resultant cone responses *E*_{_*_L_*_c,_*_M_*_c,_*_S_*_c}new_ were then transformed back to *L***a***b** coordinates via CIE tristimulus values (X, Y, and Z).
